# The possible correlation between serum GRB2 levels and carotid atherosclerosis in patients with type 2 diabetes mellitus

**DOI:** 10.3389/fendo.2022.963191

**Published:** 2022-09-13

**Authors:** Yuyan Dong, Juxiang Liu, Jing Ma, Jinxing Quan, Yanxia Bao, Yaqiang Cui

**Affiliations:** ^1^ Clinical Medical College, Ningxia Medical University, Yinchuan, China; ^2^ Department of Endocrinology, Gansu Provincial Hospital, Lanzhou, China; ^3^ The First Clinical Medical College, Lanzhou University, Lanzhou, China; ^4^ The First Clinical Medical College, Gansu University of Chinese Medicine, Lanzhou, China

**Keywords:** serum GRB2 levels, inflammatory, glycolipid metabolism, type 2 diabetes mellitus, carotid atherosclerosis

## Abstract

**Background and purpose:**

Growth factor receptor-bound protein 2(GRB2), a bridging protein. An animal study showed that downregulation of GRB2 inhibited the activation of PI3K/AKT/NF-kB pathway which improved lipid accumulation and inflammatory infiltration in rats with atherosclerosis (AS), resulting in an anti-AS effect. This was the first study to investigate blood GRB2 levels in type 2 diabetes mellitus(T2DM) patients with carotid atherosclerosis (CAS), exploring its relationship with various metabolic indicators, and further, examining whether GRB2 has an AS effect in patients with T2DM.

**Methods:**

A total of 203 participants were recruited in the study, including 69 T2DM patients without CAS (T2DM group), 67 T2DM patients with CAS (CAS group), and 67 in the age-sex-matched healthy subjects (Control group). Serum GRB2 levels were measured using enzyme-linked immunosorbent assay (ELISA) in 203 subjects who had received carotid ultrasonography. In addition, cholesterol (TC), triglycerides (TG), high-density lipoprotein cholesterol (HDL-C), low-density lipoprotein cholesterol (LDL-C), fasting plasma glucose (FPG), glycosylated hemoglobin (HBA1c), fasting insulin (FINS), hypersensitive C-reactive protein (Hs-CRP), and Interleukin 6 (IL-6) were also tested. The correlation between serum GRB2 levels and other indexes was analyzed. Finally, we analyzed the risk factors affecting carotid intima-media thickness (CIMT) in T2DM patients.

**Results:**

Serum GRB2 levels were increased in the T2DM group than in the control group, and further elevated in the CAS group (median 3.05 vs 4.40 vs 7.09 ng/ml, P<0.001). Spearman correlation analysis showed that GRB2 concentrations were negatively correlated with HDL-C, and positively associated with duration of diabetes, waist-to-hip ratio (WHR), TC, HBA1c, FPG, FINS, homeostasis model assessment-insulin resistance index (HOMA-IR), Hs-CRP, IL-6 and CIMT (P<0.01). Furthermore, serum GRB2 levels (P<0.001) remained independently related to CIMT after adjusting for the age, sex, duration of diabetes, and Body Mass Index (BMI) variables. Stepwise multiple linear regression analysis showed that IL-6, HDL-C, HBA1c, and CIMT are independent correlation factors of serum GRB2 (P<0.01). Univariate logistic regression suggested that disease duration, WHR, systolic blood pressure (SBP), TG, HDL-C, HBA1c, FPG, HOMA-IR, IL-6, Hs-CRP, and GRB2 independently associated with T2DM is combined with CAS(P<0.05). And multivariate logistic regression analysis showed that duration of diabetes, IL-6, and serum GRB2 levels were independent risk factors for T2DM combined with CAS (P<0.05), and serum GRB2 levels were a highly sensitive indicator of early AS (OR=1.405, 95% CI: 1.192-1.658 P<0.001). Moreover, the ROC curve AUC area of serum GRB2 expression levels was 0.80 (95%CI: 0.7291-0.8613, P < 0.001), with a sensitivity of 83.58% and specificity of 70.59%. The risk of CAS was substantially higher in patients with T2DM whose serum GRB2 concentration was >4.59 ng/ml.

**Conclusions:**

Serum GRB2 concentrations were significantly increased in T2DM combined with CAS, and serum GRB2 levels were linearly correlated with CIMT, suggesting that GRB2 may be involved in the occurrence and development of T2DM with CAS, which can be used as a predictor of whether T2DM is combined with CAS.

## Introduction

Diabetes is a group of chronic metabolic diseases that are characterized by persistent hyperglycemia caused by various reasons, with 90% of them being type 2 diabetes mellitus (T2DM) (1). According to the latest epidemiological data released by the International Diabetes Federation (IDF) in 2019, the number of adults with diabetes in China is 116.4 million, ranking first in the world, and is predicted to remain first in the world in the next 15 years (2). Long-term persistent hyperglycemic stimulation predisposes to various complications, and atherosclerotic cardiovascular disease is the main macrovascular complication of diabetes, which can occur in all stages of T2DM (3, 4). In recent years, although the incidence of cardiovascular and cerebrovascular disease in diabetic patients is decreasing, it is still the leading cause of death and disability in patients with T2DM (5). And carotid atherosclerosis (CAS) is partial performance of whole-body atherosclerosis and is an essential cause and early indication of ischemic stroke and transient ischemic attack (TIA) (6, 7). Using Doppler ultrasound to measure carotid intima-media thickness (CIMT) can visualize the severity of CAS and predict the risk of cardiovascular and cerebrovascular disease. Lorenz (8) et al confirmed that for each 0.1 mm thickening of the CIMT, the risk of myocardial infarction increases by 10-15%, and the risk of stroke increases by 13-18%. However, the pathogenesis of AS is complicated and still not well defined. It is crucial to identify and intervene early in sub-clinical atherosclerosis to prevent complications of macrovascular disease in patients with T2DM.

Growth factor receptor-bound protein 2 (GRB2) is a bridging protein that is normally expressed in many types of cells. Signaling pathways regulated by GRB2 are essential for maintaining numerous fundamental physiological functions such as cell proliferation, growth, and differentiation, their abnormal activation and unusual expression of GRB2 are correlated with the proliferation and invasion of cancer cells, as well as with the occurrence and development of various other chronic diseases (9, 10). Although cellular and animal experiments have found that GRB2 expression levels are associated with the accumulation of lipids, and the extent of inflammatory infiltration (11, 12). Although knockdown GRB2 has a strong anti-AS effect, further study is also needed to have a clear knowledge of its biological activity and method of action. To investigate the clinical importance of GRB2 in humans, we tested plasma GRB2 concentrations in healthy control recipients, patients with T2DM, and patients with T2DM coupled with CAS, and evaluated the relationship between GRB2 concentrations and anthropometric and metabolic variables.

## Materials and methods

### Research subjects

From March to December 2021, 136 diabetic patients (aged 35-65 years) who attended the endocrinology department of Gansu Provincial Hospital were enrolled in our study, of which 69 patients had T2DM without CAS and 67 patients had T2DM coupled with CAS. CIMT thickening (CIMT ≥ 0.9 mm) or carotid plaque was employed as the criteria for the diagnosis of CAS according to ultrasound detection. In addition, 67 age-sex-matched subjects were examined at the same period. The exclusion criteria are as follows: Other types of diabetes outside T2DM; serious acute and chronic diabetic complications. Other system disorders including severe liver and renal insufficiency, malignant tumors, and cardiovascular diseases such as hypertension, coronary heart disease, and cerebral infarction. Stressful situations such as infection, surgery, or trauma. Usage of thiazolidinediones hypoglycemic medicines, lipid regulating agents, angiotensin-converting enzyme inhibitors, and hormonal drugs within the recent 3 months. To rule out long-term smokers and drinkers, each subject was rigorously questioned about his or her smoking and drinking history. Patients and family members refused to cooperate. The study was approved by the Clinical Research Ethics Committee of Gansu Provincial Hospital. Informed consent was given to each participant.

### Anthropometric and biochemical measurements

General anthropometric indicators for all subjects such as age, sex, height, weight, waist circumference, hip circumference, history of smoking, history of drinking, systolic blood pressure (SBP), diastolic blood pressure (DBP), and duration of diabetes were all collected and recorded, BMI (weight(kg)/the square of height(m)) and WHR (the ratio of waist circumference to hip circumference) were calculated. The commissioner of the Laboratory Department of Gansu Provincial Hospital was responsible for testing biochemical indicators such as cholesterol (TC), triglycerides (TG), high-density lipoprotein cholesterol (HDL-C), low-density lipoprotein cholesterol (LDL-C), glycated hemoglobin (HBA1c), fasting plasma glucose (FPG), fasting insulin (FINS), hypersensitive C-reactive protein (Hs-CRP) and interleukin-6 (IL-6). The hepatic insulin resistance was determined by the homeostasis model assessment (HOMA): HOMA-IR=FPG×FINS/22.5. Carotid ultrasound (Siemens Acuson×300 and 7.5 MHz frequency color Doppler ultrasound, Germany) was examined by a specialized sonographer in all subjects to identify CIMT. Specific testing techniques were meticulously followed in accordance with norms.

### Determination of serum GRB2 concentration

Five milliliters of venous blood were collected from each fasting patient and promptly centrifuged at 4°C for 10 minutes at 3000r/min, after which the serum specimens were separated and labeled before being kept in a -80°C refrigerator. Finally, after collecting all of the materials, the GRB2 concentration was measured evenly. The concentration of GRB2 in plasma was evaluated using the Human GRB2 ELISA kit as directed by the manufacturer (JL Biologicals). ELISA has a detection range of 78-5000pg/ml. The kit has a minimum detection concentration of less than 0.1ng/ml, an intra-plate coefficient of variation of less than 9%, and an inter-plate coefficient of variation of less than 11%. An enzyme analyzer was used to measure the absorbance at 450 nm (OD) (RT-6500). A standard curve was produced using the standard concentration (X) and the standard OD at 450 nm (Y), and the standard curve was fitted using a logistic equation, and the sample concentration was calculated.

### Statistical analysis

The test results were analyzed using IBM SPSS23.0 and GraphPad Prism 8.0. Continuous variables that had a normal distribution were expressed as mean standard deviation (X ± SD), continuous variables that did not have a normal distribution were expressed as median and interquartile spacing (M (P25, P75)), and categorical variables were expressed as percentages. Before proceeding with the study, non-normally distributed variables were subjected to a natural logarithmic transformation. In the three groups, one-way ANOVA was employed to measure data that fit a normal distribution and a log-transformed normal distribution. For those that did not fit to a normal distribution, a non-parametric K-W test (Kruskal-Wallis test) was performed, a K-W one-way ANOVA test was utilized for group comparison, and a χ^2^ test was used to compare differences in categorical variables across the three groups. Spearman correlation was employed to assess the relationship between the variables. Correlations were evaluated using partial correlation after the effects of age, gender, diabetes duration, and BMI were adjusted. The impacts of different variables were calculated using univariate regression analysis, and then multiple regression models were built. Making receiver operating characteristic (ROC) curves to assess diagnostic performance. P < 0.05 showed that the difference was statistically significant in all of the various statistical approaches.

## Results

### General information of the three groups of patients

**Table 1** shows the general information of three groups of patients. There was no significant difference in age, sex, smoking history, drinking history, BMI, SBP, DBP and there was no statistical difference in the duration of diabetes between the T2DM group and the CAS group (p>0.05). In addition, as compared to the Control group, WHR was greater in the CAS group (p<0.01). The CAS group was outperformed than the T2DM group and the Control group in terms of CIMT (P < 0.001) (**Table 1**).

**Table 1:** General information of the Control group, the T2DM group, and the CAS group.

Parameters	Control Group	T2DM group	CAS group	p-value
	(n=67)	(n=69)	(n=67)	
Age(years)	49.67±5.82	50.91±7.36	51.84±5.06	0.128
Sex (male)	38(56.7%)	39(58.2%)	46(68.7%)	0.256
BMI (kg/m^2^)	24.31±2.31	24.20±3.14	24.39±2.95	0.615
WHR	0.90(0.86,0.95) ^c^	0.92(0.87,0.97)	0.95(0.90,0.99) ^a^	<0.01
Disease course(years)		3.0(0.5-8.0)	4.0(1.0-7.0)	1.000
SBP (mm/Hg)	121.22±11.75	122.92±12.26	125.99±13.81	0.089
DBP (mm/Hg)	78.06±7.79	78.58±9.29	81.00±9.17	0.120
Smoking	24(35.8%)	23(33.3%)	27(40.3%)	0.694
Alcohol	26(38.8%)	14(20.3%)	23(34.3%)	0.051
CIMT (mm)	0.68(0.60,0.74) ^c^	0.69(0.63,0.75) ^c^	0.95(0.90,1.05) ^a, b^	<0.001

a, significant p<0.05 vs. the Control group; b, significant p<0.05 vs. the T2DM group; c, significant p<0.05 vs. the CAS group.

### Biochemical characteristics of the three groups of patients

**Figure 1:**
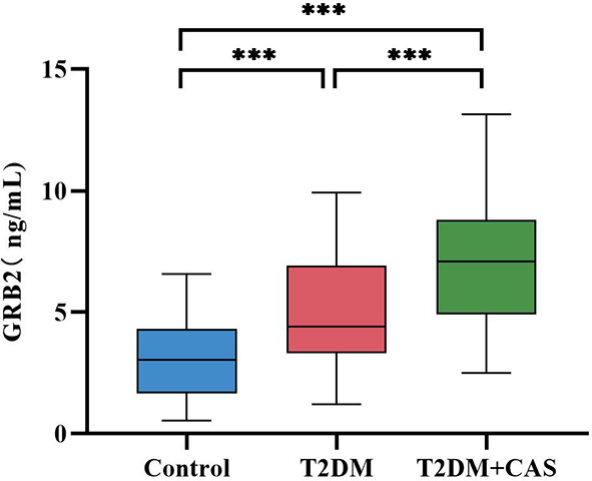
Comparison of the Serum GRB2 levels between the Control group, the T2DM group, and the CAS group. The differences were statistically significant. ***:P<0.001.

The biochemical features of the three groups are summarized in **Table 2**. There was no significant difference in TC and LDL-C between the Control group, the T2DM group, and the CAS group(p>0.05). Compared with the Control group, TG in the CAS group was increased (p<0.05), HBA1c, FPG, HOMA-IR, and Hs-CRP in the T2DM group, and the CAS group were increased (P < 0.05), HDL-C in the T2DM group and the CAS group were lower (P < 0.001), while the differences between the T2DM group and the CAS group did not reach statistical significance (P > 0.05). The T2DM group was greater than the Control group when compared to FINS (P < 0.05). Meaningfully, IL-6 (median 1.34 vs 2.07 vs 2.91pg/ml, P < 0.001) and serum GRB2 levels (median 3.05 vs 4.40 vs 7.09, P < 0.001ng/ml) (**Figure 1**) were significantly higher in the T2DM group than the healthy population and further increased in the CAS patients (P<0.001) (**Table 2**).

**Table 2:** Biochemical characteristics of the Control group, the T2DM group, and the CAS group.

Parameters	Control group	T2DM group	CAS group	p-value
	(n=67)	(n=69)	(n=67)	
TC (mmol/L)	4.59(4.22,5.22)	4.62(3.94,5.41)	4.57(3.94,5.39)	0.512
TG (mmol/L)	1.30(1.02,1.81) ^c^	1.67(1.14,2,26)	1.71(1.24,1.81) ^a^	<0.01
HDL-C(mmol/L)	1.24(1.05,1.41) ^b, c^	0.97(0.88,1.17^a^	0.96(0.82,1.12) ^a^	<0.001
LDL-C(mmol/L)	2.65(2.30,2.90)	2.82(2.43,3.37)	2.62(2.04,3.10)	0.215
HBA1c (%)	5.44±0.31 ^b, c^	9.39±2.15^a^	8.91±2.18^a^	<0.001
FPG (mmol/L)	4.97(4.76,5.26) ^b, c^	10.45(7.28,12.88) ^a^	9.02(7.53,13.63) ^a^	<0.001
FINS (μU/mL)	5.90(4.70,7.50) ^b^	7.90(4.10,13.00) ^a^	6.60(5.30,9.70)	<0.05
HOMA-IR	1.35(1.02,1.67) ^b, c^	3.30(1.87,5.03) ^a^	3.09(2.20,4.29) ^a^	<0.001
Hs-CRP (mg/L)	0.70(0.30,1.10) ^b, c^	1.30(0.60,3.85) ^a^	1.20(0.70,3.00) ^a^	<0.001
IL-6(pg/ml)	1.34(1.03,1.64) ^b, c^	2.07(1.47,2.97) ^a, c^	2.91(1.91,4.05) ^a, b^	<0.001
GRB2(ng/ml)	3.05(1.65,4.31) ^b, c^	4.40(3.32,6.91) ^a, c^	7.09(4.91,8.80) ^a, b^	<0.001

a, significant p<0.05 vs. the Control group; b, significant p<0.05 vs. the T2DM group; c, significant p<0.05 vs. the CAS group.

### Correlation of serum GRB2 levels with various clinical indicators

**Figure 2:**
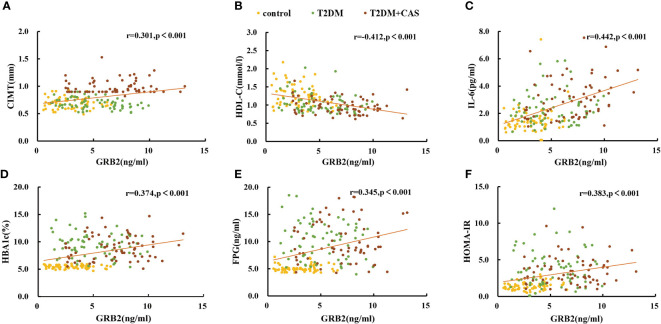
Scatter plots showing the correlation between serum GRB2 concentrations and **(A)** CIMT, **(B)** HDL-C, **(C)** IL-6, **(D)** HBA1c, **(E)** FPG, and **(F)** HOMA-IR in subjects.

Serum GRB2 concentrations were shown to be negatively connected with HDL-C, but positively correlated with illness duration, WHR, TC, HBA1c, FPG, FINS, HOMA-IR, Hs-CRP, IL-6, and CIMT (P<0.01) (**Table 3**; **Figure 2**). After adjusting for age, gender, illness duration, and BMI, CIMT, HDL-C, IL-6, Hs-CRP, HBA1c, FPG, and HOMA-IR (P<0.01) remained independently linked with serum GRB2 (**Table 3**). Because of the number of related variables and considering the problem of multicollinearity, we chose a stepwise multiple linear regression model to assess the predictors of elevated GRB2. In the regression analysis results, Durbin-Watson (D-W) value =1.796, indicating that there was no autocorrelation between variables and the model was well constructed. In addition, the standardized residuals have a mean of 0 and a standard deviation of 0.99, which is close to 1 (the standardized residuals are close to the Standard normal distribution). Therefore, we concluded that IL-6, HDL-C, HBA1c, and CIMT are independent serum GRB2 correlation variables (**Table 4**).

**Table 3:** Correlation analysis of variables associated with serum GRB2 concentration in the study population.

Parameters	GRB2		GRB2	
	(unadjusted)		(age, sex, disease course, and BMI adjusted)	
	r value	p-value	r value	p-value
Disease course	0.400	0.000	~	~
Age	-0.075	0.285	~	~
Sex	-0.127	0.072	~	~
BMI	0.107	0.127	~	~
WHR	0.226	0.001	0.071	0.317
Smoking	0.106	0.133	0.063	0.373
Alcohol	-0.014	0.845	-0.074	0.301
SBP	0.022	0.752	-0.006	0.928
DBP	0.125	0.076	0.129	0.070
TC	-0.155	0.027	-0.114	0.108
TG	0.129	0.067	0.093	0.191
HDL-C	-0.412	0.000	-0.336	0.000
LDL-C	-0.008	0.907	0.009	0.902
HBA1c	0.374	0.000	0.268	0.000
FPG	0.345	0.000	0.219	0.002
FINS	0.214	0.002	0.137	0.054
HOMA-IR	0.383	0.000	0.217	0.002
Hs-CRP	0.287	0.000	0.273	0.000
IL-6	0.442	0.000	0.400	0.000
CIMT	0.301	0.000	0.332	0.000

**Table 4:** Stepwise multiple linear regression analysis with GRB2 as the dependent variable.

Independent variable	B	Beta	SE	t	P	VIF
(Constant)	2.584		1.227	2.106	0.036	
IL-6 (pg/ml)	0.455	0.280	0.101	4.497	<0.001	1.122
HDL-C (mmol/L)	-1.977	-0.227	0.552	-3.582	<0.001	1.161
HBA1c (%)	0.185	0.170	0.068	2.742	0.007	1.113
CIMT (mm)	2.614	0.170	0.970	2.693	0.008	1.154

The following independent variables were considered for the model: duration of diabetes, WHR, HDL-C, HBA1c, FPG, FINS, Hs-CRP, HOMA-IR, IL-6, and GRB2. Only the variables that had a p<0.05 were considered in the final fitted model.

### Correlation of T2DM combined CAS with various factors

**Figure 3:**
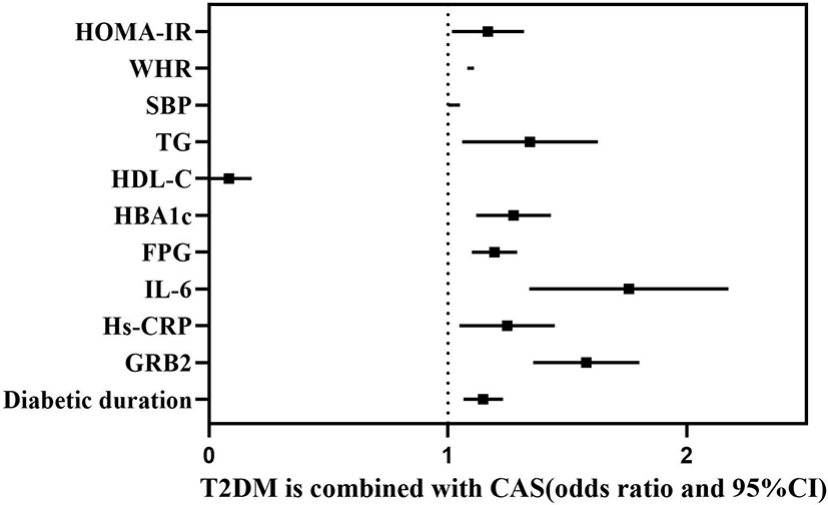
Forest plot showing the influencing factors associated with whether T2DM is combined with CAS.

**Figure 4:**
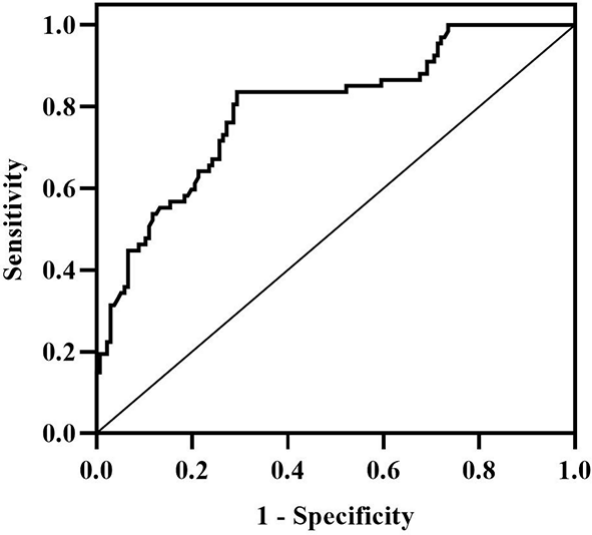
ROC curve.

According to Spearman’s correlation study, CIMT was inversely connected with HDL-C and females compared to men, but favorably correlated with age, illness duration, WHR, SBP, DBP, HBA1C, FPG, HOMA-IR, Hs-CRP, IL-6, and GRB2 (P<0.05) (**Table 5**). And after controlling for age, gender, illness duration, and BMI, GRB2, HDL-C, IL-6, Hs-CRP, HBA1c, DBP, and FPG remained independently linked with CIMT (P<0.05) (**Table 5**). To investigate further the risk variables influencing CIMT, we conducted a binary logistic regression analysis of all covariates with whether CIMT is thickened as the dependent variable. A univariate logistic regression analysis indicated that disease duration, WHR, SBP, TG, HDL-C, HBA1c, FPG, HOMA-IR, IL-6, Hs-CRP, and GRB2 independently associated with T2DM is combined with CAS(P<0.05) (**Table 6**; **Figure 3**). And multivariate logistic regression analysis showed that disease duration, IL-6, and serum GRB2 levels were independent risk factors for T2DM combined with CAS (P<0.05), and serum GRB2 levels were a highly sensitive indicator of early AS (OR=1.405, 95% CI: 1.192-1.658 P<0.001) (**Table 7**). In addition, the area under the ROC curve was 0.80 (95%CI: 0.7291-0.8613, P<0.001), the best diagnostic threshold was 4.59 ng/ml, the sensitivity was 83.58%, and the specificity was 70.59%, indicating that serum GRB2 has a high diagnostic value for T2DM with CAS (**Figure 4**). In conclusion, serum GRB2 may be utilized as a screening biomarker for T2DM with CAS.

**Table 5:** Correlation analysis of variables associated with CIMT in the study population.

Parameters	CIMT		CIMT	
	(unadjusted)		(age, sex, disease course, and BMI adjusted)	
	r value	p-value	r value	p-value
Disease course	0.367	0.000	~	~
Age	0.282	0.000	~	~
Sex	-0.147	0.037	~	~
BMI	0.025	0.719	~	~
WHR	0.203	0.004	0.116	0.102
Smoking	0.084	0.236	0.066	0.351
Alcohol	0.103	0.145	0.046	0.518
SBP	0.228	0.001	0.127	0.073
DBP	0.15	0.033	0.140	0.049
TC	-0.106	0.133	0.088	0.216
TG	0.071	0.312	0.125	0.077
HDL-C	-0.303	0.000	-0.271	0.000
LDL-C	-0.059	0.400	-0.069	0.332
HBA1c	0.232	0.001	0.164	0.021
FPG	0.275	0.000	0.197	0.005
FINS	0.056	0.430	-0.02	0.774
HOMA-IR	0.234	0.001	0.082	0.249
Hs-CRP	0.178	0.011	0.185	0.009
IL-6	0.395	0.000	0.191	0.007
GRB2	0.301	0.000	0.332	0.000

**Table 6:** Correlation of serum GRB2 levels with T2DM combined with CAS (univariate logistic regression).

Parameters	OR	OR 95% CI	P-value
**Age(years)**	1.042	0.992-1.093	0.099
**Sex**	1.678	0.905-3.113	0.100
**Disease duration(years)**	1.142	1.067-1.223	<0.001
**BMI (kg/m^2^)**	1.050	0.951-1.158	0.334
**WHR**	1.095	1.081-1.109	0.004
**SBP (mm/Hg)**	1.025	1.001-1.050	0.042
**DBP (mm/Hg)**	1.034	1.000-1.070	0.052
**Smoking**	0.782	0.428-1.429	0.425
**Drinking**	0.797	0.427-1.489	0.477
**TC (mmol/L)**	0.815	0.592-1.122	0.210
**TG (mmol/L)**	1.323	1.069-1.637	<0.05
**HDL-C(mmol/L)**	0.045	0.011-0.191	<0.001
**LDL-C(mmol/L)**	1.098	1.017-1.185	0.195
**HBA1c (%)**	1.268	1.122-1.434	<0.001
**FPG (mmol/L)**	1.192	1.101-1.290	<0.001
**FINS (μU/mL)**	1.005	0.946-1.067	0.880
**HOMA-IR**	1.160	1.019-1.321	0.025
**IL-6 (pg/mL)**	1.724	1.358-2.190	<0.001
**Hs-CRP (mg/L)**	1.237	1.053-1.452	0.009
**GRB2 (ng/ml)**	1.568	1.361-1.807	<0.001

All variables were considered for a one-way logistic regression analysis: age, sex, duration of diabetes, BMI,WHR,SBP,DBP,smoking,dringing,TG,HDL-C,LDL-C,HBA1c,FPG,INS,HOMA-IR,Hs-CRP,IL-6 and GRB2.OR, odds ratio; CI, confidence interval.

**Table 7:** Multivariate logistic regression analysis with whether CIMT is thickened as the dependent variable.

Parameters	OR	OR 95% CI	P-value
Disease duration(years)	1.093	1.003-1.192	0.043
IL-6 (pg/mL)	1.327	1.009-1.745	0.043
GRB2 (ng/ml)	1.405	1.192-1.658	<0.001

All variables were considered for one-way logistic regression analysis, only the variables that had a p<0.05 were considered in a multi-factor logistic regression analysis.

OR, odds ratio; CI, confidence interval.

## Discussion

GRB2 levels fluctuate in relation to glucolipid metabolism and the inflammatory response. However, it has not been documented if serum GRB2 changes in T2DM when paired with CAS, nor has the link between GRB2 and diabetic macroangiopathy. In this study, we discovered that blood GRB2 levels in T2DM patients were higher than in the healthy population for the first time, and that they were much higher following combined with CAS. Furthermore, serum GRB2 concentration was found to be positively linked with CIMT and to be an independent risk factor for combined CAS in T2DM.

The pathogenesis of AS is complicated and still not well defined, and lipid deposition, as well as inflammatory response, are two very important aspects of it (13). More and more research in recent years have revealed that the PI3K/AKT/NF-kB pathway is intimately linked to the development of AS. The PI3K/AKT/NF-kB pathway is involved in a variety of functions in the artery wall, including lipid metabolism, smooth muscle, fibroblast proliferation, and collagen synthesis. The NF-kB pathway is also an essential inflammation-related mechanism in the etiology of AS (14). In addition, the mitogen-activated protein kinase (MAPK) pathway, which regulates the proliferation and migration of vascular endothelial cells and smooth muscle cells, is also implicated in the etiology of AS (15). Shen Tong (12) et al demonstrated that GRB2 knockdown inhibited the activation of the PI3K/AKT/NF-kB pathway, which reduced lipid accumulation and inflammatory infiltration in AS rats and exerted an against effect in AS, and that downregulation of GRB2 expression significantly reduced CRP and tumor necrosis factor (TNF-α) levels in the peripheral blood of AS rats. On the side, GRB2 is a key component upstream of the MAPK signaling pathway, and cell culture experiments using mouse bone marrow-derived macrophages have demonstrated that oxidized low-density lipoprotein (Ox-LDL)-induced MAPK pathway activation and foam cell production need GRB2 participation (11, 16). Besides, dysregulation of IL-6 activity is linked to chronic inflammation. Experiments on animals revealed that injecting high amounts of IL-6 into male mice fed a normal or high-fat diet increased the onset and progression of AS (17). Hs-CRP is mainly released induced by IL-6 in the liver and is not detectable in healthy arteries, nonetheless, it is identified in the early stages of AS and accumulates as the disease progresses, it is considered a predictor of future cardiovascular events (18, 19). This research found that blood GRB2 and IL-6 levels were considerably greater in patients with T2DM with CAS than in patients with T2DM without CAS, and that GRB2 was closely linked with HDL-C, IL-6, and Hs-CRP, which was similar with the findings of earlier animal model studies. As a result, GRB2 may be implicated in lipid metabolism and inflammatory response *via* the aforementioned processes, playing a crucial role in the development of combined CAS in T2DM patients. Nevertheless, it is unknown if lipid-affecting medicines influence serum GRB2 levels. Subjects who had taken hormones or oral lipid-lowering medicines during the previous three months were excluded from our investigation, avoiding possible drug-induced confounding effects and boosting the reliability of the study’s findings. Additionally, numerous additional pro-inflammatory cytokines, such as IL-8, tumor necrosis factor (TNF-α), and monocyte chemotactic protein-1 (MCP-1) have been linked to chronic inflammation in AS (20, 21). Therefore, additional pro-inflammatory cytokines need to be detected to further assess the correlation between GRB2 and chronic inflammation in AS. In conclusion, more research is needed to understand how GRB2 regulates metabolism and how it interacts with cardiovascular risk factors.

GRB2 may also have a role in the establishment and progression of T2DM by binding to phosphorylated insulin receptor substrate 1 (IRS1) and subsequently regulating insulin levels *via* RAS activation of the MAPK pathway (22). The MAPK signaling pathway can also increase the expression level of IR by acting on the transcription factor ETS-1 to regulate insulin sensitivity and glucose stability (23). It has been shown that the expression levels of GRB2 were significantly upregulated in T2DM mice and cellular models (24). Consistent with their report, we also found clues to a potential role of GRB2 in the regulation of glucose metabolism and insulin resistance, the results of this study showed that serum GRB2 levels were significantly higher in the T2DM group than in healthy controls, and serum GRB2 levels correlated with the duration of diabetes, HBA1c, FPG, and HOMA-IR. It was previously shown that increased phosphorylation of insulin receptor substrate 2 (IRS2) serine in a hyperglycemic state decreased GRB2 bridging protein expression in diabetic neuropathy rats, whereas GRB2 expression was increased in diabetic retinopathy mice because it bound to phosphorylated insulin receptor substrate 1 (IRS1), activated the MAPK signaling pathway, and was also involved in vascular endothelial growth factor signaling to lead to retraction (25–27). Significantly, we included T2DM patients excluding subjects with severely acute and chronic complications of T2DM, and were able to better investigate the factors influencing serum GRB2 in a purely hyperglycemic state. However, more studies are needed to further clarify the role and potential mechanisms of GRB2 in glucose metabolism.

The limitations of our study are also worth commenting on. First of all, because this was a cross-sectional investigation, it was unable to conclude a causal association between elevated blood GRB2 levels and the development of T2DM paired with CAS. Furthermore, all research participants were drawn from a single province, and the population was underrepresented. As a result, a large-scale, prospective study with a high sample size might be done in the future to confirm the link between blood GRB2 levels and T2DM patients with CAS. Third, our work was based on a single blood measurement, which may not accurately represent serum GRB2 concentrations throughout time, and serum GRB2 concentrations should be assessed at different phases to better understand its function in the pathogenesis of T2DM and T2DM with CAS.

## Conclusions

Serum GRB2 levels were higher in T2DM patients compared to the healthy population, and they were much higher following the addition of CAS, and there was a strong association between serum GRB2 and CIMT. Elevated blood GRB2 concentrations may be an independent risk factor for CAS in T2DM patients and can be utilized as a predictor of CAS in T2DM patients. Furthermore, serum GRB2 concentrations are linked to glucolipid metabolism, inflammatory variables, and insulin resistance, and more research is needed in the future to determine the possible pathophysiological function of GRB2 in T2DM with CAS.

## Data availability statement

The raw data supporting the conclusions of this article will be made available by the authors, without undue reservation.

## Ethics statement

The studies involving human participants were reviewed and approved by Clinical Research Ethics Committee of Gansu Provincial Hospital, Lanzhou, China. The patients/participants provided their written informed consent to participate in this study.

## Author contributions

YD and JL designed the research, analyzed the data and wrote the manuscript. JM interpreted and revised the manuscript. JQ provided the platform and funding for the research and participated in guiding the entire research process. YB and YC recruited subjects and collected the data. All authors contributed to the article and approved the submitted version.

## Funding

This study was supported by the National Natural Science Foundation of China (81860091) and the Research project of the People’s Hospital of Gansu Province(16GSSY1-1).

## Conflict of interest

The authors declare that the research was conducted in the absence of any commercial or financial relationships that could be construed as a potential conflict of interest.

## Publisher’s note

All claims expressed in this article are solely those of the authors and do not necessarily represent those of their affiliated organizations, or those of the publisher, the editors and the reviewers. Any product that may be evaluated in this article, or claim that may be made by its manufacturer, is not guaranteed or endorsed by the publisher.
