# Corrigendum: The possible correlation between serum GRB2 levels and carotid atherosclerosis in patients with type 2 diabetes mellitus

**DOI:** 10.3389/fendo.2022.1046738

**Published:** 2022-10-21

**Authors:** Yuyan Dong, Juxiang Liu, Jing Ma, Jinxing Quan, Yanxia Bao, Yaqiang Cui

**Affiliations:** ^1^ Clinical Medical College, Ningxia Medical University, Yinchuan, China; ^2^ Department of Endocrinology, Gansu Provincial Hospital, Lanzhou, China; ^3^ The First Clinical Medical College, Lanzhou University, Lanzhou, China; ^4^ The First Clinical Medical College, Gansu University of Chinese Medicine, Lanzhou, China

**Keywords:** serum GRB2 levels, inflammatory, glycolipid metabolism, type 2 diabetes mellitus, carotid atherosclerosis

In the published article, there was an error in **Table 1** as published. The p-value for the Disease course in **Table 1** is written as 1000, which should have been recorded as 1.000, as in the original data submitted. The corrected **Table 1** and its caption **General information of the Control group, the T2DM group, and the CAS group appear below.

**Table 1:** General information of the Control group, the T2DM group, and the CAS group.

Parameters	Control group (n=67)	T2DM group (n=69)	CAS group (n=67)	p-value
Age(years)	49.67 ± 5.82	50.91 ± 7.36	51.84 ± 5.06	0.128
Sex (male)	38(56.7%)	39(58.2%)	46(68.7%)	0.256
BMI (kg/m^2^)	24.31 ± 2.31	24.20 ± 3.14	24.39 ± 2.95	0.615
WHR	0.90(0.86,0.95)^c^	0.92(0.87,0.97)	0.95(0.90,0.99)^a^	<0.01
Disease course(years)		3.0(0.5-8.0)	4.0(1.0-7.0)	1.000
SBP (mm/Hg)	121.22 ± 11.75	122.92 ± 12.26	125.99 ± 13.81	0.089
DBP (mm/Hg)	78.06 ± 7.79	78.58 ± 9.29	81.00 ± 9.17	0.120
Smoking	24(35.8%)	23(33.3%)	27(40.3%)	0.694
Alcohol	26(38.8%)	14(20.3%)	23(34.3%)	0.051
CIMT (mm)	0.68(0.60,0.74)^c^	0.69(0.63,0.75)^c^	0.95(0.90,1.05)^a,b^	<0.001

^a^significant p<0.05 vs. the Control group; ^b^significant p<0.05 vs. the T2DM group; ^c^significant p<0.05 vs. the CAS group.

In the published article, there was an error in **Figure 2** as published. In the scatter plot of GRB2 and FPG, the unit of FPG is written as ng/ml instead of mmol/l. The corrected **Figure 2** and its caption **Scatter plots showing the correlation between serum GRB2 concentrations and (a)CIMT, (b)HDL-C, (c)IL-6, (d)HBA1c, (e)FPG, and (f)HOMA-IR in subjects appear below.

**Figure 2:**
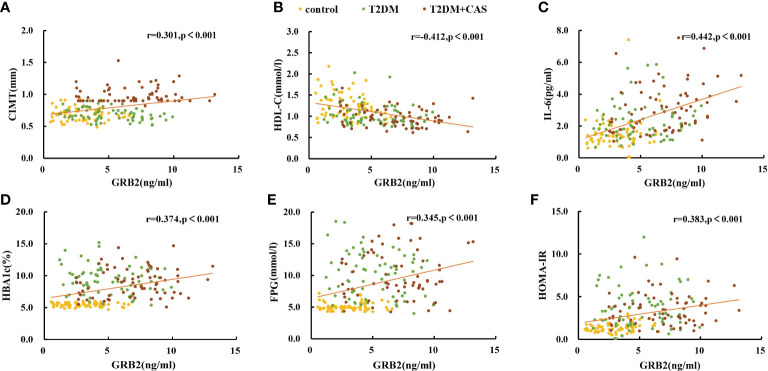
Scatter plots showing the correlation between serum GRB2 concentrations and **(A)** CIMT, **(B)** HDL-C, **(C)** IL-6, **(D)** HBA1c, **(E)** FPG, and **(F)** HOMA-IR in subjects.

In the published article, there was an error. DBP was erroneously included within the text.

A correction has been made to **Results**, *Correlation of T2DM combined CAS with various factors*, Paragraph Number 1. This sentence previously stated:

“A univariate logistic regression analysis indicated that disease duration, WHR, SBP, TG, HDL-C, HBA1c, DBP, FPG, HOMA-IR, IL-6, Hs-CRP, and GRB2 independently associated with T2DM is combined with CAS(P<0.05)”

The corrected sentence appears below:

“A univariate logistic regression analysis indicated that disease duration, WHR, SBP, TG, HDL-C, HBA1c, FPG, HOMA-IR, IL-6, Hs-CRP, and GRB2 independently associated with T2DM is combined with CAS(P<0.05)”

The authors apologize for these error and state that they do not change the scientific conclusions of the article in any way. The original article has been updated.

## Publisher’s note

All claims expressed in this article are solely those of the authors and do not necessarily represent those of their affiliated organizations, or those of the publisher, the editors and the reviewers. Any product that may be evaluated in this article, or claim that may be made by its manufacturer, is not guaranteed or endorsed by the publisher.

